# Supplementary material for: The Post-Traumatic Stress Disorder Checklist for DSM-5: Psychometric Properties of the Italian Version
Source: Int J Environ Res Public Health. 2022 Apr 26;19(9):5282. doi: 10.3390/ijerph19095282 (PMC9105570; doi:10.3390/ijerph19095282)
Supplement: Supplementary file 1 [file ijerph-19-05282-s001.zip › ijerph-1658536-supplementary.pdf]

Table S1. Pearson's correlations among PCL items.

Item	1	2	3	4	5	6	7	8	9	10	11	12	13	14	15	16	17	18	19
2	.68																		
3	.58	.51																	
4	.66	.52	.64																
5	.57	.49	.63	.68															
6	.57	.46	.50	.60	.54														
7	.53	.47	.52	.50	.48	.63													
8	.30	.32	.31	.32	.34	.38	.42												
9	.42	.44	.41	.43	.39	.42	.41	.30											
10	.39	.38	.45	.40	.37	.44	.44	.32	.70										
11	.60	.50	.55	.59	.55	.52	.50	.35	.67	.67									
12	.54	.52	.49	.49	.49	.49	.52	.31	.53	.51	.62								
13	.46	.41	.44	.44	.43	.42	.47	.35	.59	.53	.60	.67							
14	.51	.48	.47	.51	.47	.46	.45	.29	.60	.52	.59	.69	.68						
15	.51	.45	.47	.46	.46	.46	.47	.27	.49	.51	.59	.61	.62	.60					
16	.39	.41	.44	.34	.41	.37	.35	.31	.44	.42	.43	.45	.46	.49	.47				
17	.52	.45	.47	.52	.46	.49	.47	.33	.43	.41	.60	.50	.50	.51	.50	.44			
18	.54	.48	.47	.56	.53	.51	.48	.33	.56	.51	.68	.56	.60	.60	.56	.43	.69		
19	.56	.46	.48	.54	.52	.56	.46	.32	.48	.46	.59	.63	.59	.64	.61	.46	.55	.66	
20	.52	.53	.45	.47	.49	.45	.44	.30	.43	.39	.50	.58	.51	.56	.52	.46	.50	.53	.60

Note: all correlations are significant at $p < .01$. A higher color intensity indicates a stronger correlation.

Table S2. Factor loadings for the 7-factor structure model.

	Factor						
**Item**	**1. Re-experiencing**	**2. Avoidance**	**3. Negative affect**	**4. Anhedonia**	**5. Externalized behavior**	**6. Anxious arousal**	**7. Dysphoric arousal**
1	0.811						
2	0.705						
3	0.757						
4	0.821						
5	0.769						
6		0.821					
7		0.766					
8			0.428				
9			0.795				
10			0.778				
11			0.875				
12				0.828			
13				0.812			

14	0.834			
15		0.773		
16		0.611		
17			0.779	
18			0.885	
19				0.822
20				0.726

Note: p is <.001 for all factor loadings.
